# Adapting the Number of Questions Based on Detected Psychological Distress for Cognitive Behavioral Therapy With an Embodied Conversational Agent: Comparative Study

**DOI:** 10.2196/50056

**Published:** 2024-03-14

**Authors:** Kazuhiro Shidara, Hiroki Tanaka, Hiroyoshi Adachi, Daisuke Kanayama, Takashi Kudo, Satoshi Nakamura

**Affiliations:** 1 Nara Institute of Science and Technology Ikoma Japan; 2 Health and Counseling Center Osaka University Toyonaka Japan

**Keywords:** cognitive behavioral therapy, psychological distress detection, embodied conversational agents, automatic thoughts, long short-term memory, multitask learning

## Abstract

**Background:**

The high prevalence of mental illness is a critical social problem. The limited availability of mental health services is a major factor that exacerbates this problem. One solution is to deliver cognitive behavioral therapy (CBT) using an embodied conversational agent (ECA). ECAs make it possible to provide health care without location or time constraints. One of the techniques used in CBT is Socratic questioning, which guides users to correct negative thoughts. The effectiveness of this approach depends on a therapist’s skill to adapt to the user’s mood or distress level. However, current ECAs do not possess this skill. Therefore, it is essential to implement this adaptation ability to the ECAs.

**Objective:**

This study aims to develop and evaluate a method that automatically adapts the number of Socratic questions based on the level of detected psychological distress during a CBT session with an ECA. We hypothesize that this adaptive approach to selecting the number of questions will lower psychological distress, reduce negative emotional states, and produce more substantial cognitive changes compared with a random number of questions.

**Methods:**

In this study, which envisions health care support in daily life, we recruited participants aged from 18 to 65 years for an experiment that involved 2 different conditions: an ECA that adapts a number of questions based on psychological distress detection or an ECA that only asked a random number of questions. The participants were assigned to 1 of the 2 conditions, experienced a single CBT session with an ECA, and completed questionnaires before and after the session.

**Results:**

The participants completed the experiment. There were slight differences in sex, age, and preexperimental psychological distress levels between the 2 conditions. The adapted number of questions condition showed significantly lower psychological distress than the random number of questions condition after the session. We also found a significant difference in the cognitive change when the number of questions was adapted based on the detected distress level, compared with when the number of questions was fewer than what was appropriate for the level of distress detected.

**Conclusions:**

The results show that an ECA adapting the number of Socratic questions based on detected distress levels increases the effectiveness of CBT. Participants who received an adaptive number of questions experienced greater reductions in distress than those who received a random number of questions. In addition, the participants showed a greater amount of cognitive change when the number of questions matched the detected distress level. This suggests that adapting the question quantity based on distress level detection can improve the results of CBT delivered by an ECA. These results illustrate the advantages of ECAs, paving the way for mental health care that is more tailored and effective.

## Introduction

### Background

Cognitive behavioral therapy (CBT) is an established and effective therapeutic approach for treating a wide range of mental illnesses, including depression and anxiety disorder [1,2]. This approach, rooted in the cognitive model of emotional responses, posits that our thoughts, feelings, and behaviors are interconnected. Its central principle asserts that negative automatic thoughts must be corrected because they deleteriously affect emotions and behaviors [3]. CBT has been widely adopted in clinical practice and in preventive health care for the general public [2,4]. Despite its efficacy, broad dissemination faces challenges [5,6]. The World Health Organization [5] asserts that factors such as a shortage of trained therapists, geographical and financial limitations, and societal stigma toward mental health contribute to a substantial treatment gap.

Researchers have attempted to provide CBT through embodied conversational agents (ECAs) to address social issues [7-9]. ECAs are computer-programmed interfaces that simulate human-like conversations with users by using natural language processing techniques. Examples include messaging app–like chatbots [10,11], robots [12,13], and ECAs displayed as computer graphics on a screen [14]. Text-based agents or chatbots are especially widespread and commercially available as smartphone apps. Inkster et al [11] and Fitzpatrick et al [10] are 2 examples of such agents whose ability has been extensively validated for targeting and helping individuals who are experiencing mild to moderate symptoms of depression and anxiety. These agents provide therapy for daily mental health care rather than intensive clinical treatment. Studies have shown their effectiveness in reducing symptoms of depression and anxiety, increasing mental well-being, and fostering user resilience [15].

ECAs, or chatbots, increase accessibility, affordability, and anonymity, potentially reaching a broader population that might benefit from CBT. However, the effectiveness of these digital therapies depends on several factors, including therapeutic techniques, empathy, user interface, and personalization. Asay and Lambert [16] categorized the factors affecting therapy effectiveness into four major groups: (1) extratherapeutic changes, which include clients’ personality and environmental factors that support recovery, independent of therapy participation; (2) common factors such as empathy and therapeutic alliance, which are found in various therapy approaches; (3) expectancy, which involves improvement resulting from clients’ anticipation of assistance and their belief in the therapy’s rationale and effectiveness; and (4) techniques, which are specific factors unique to particular therapies and adjusted to address specific issues. Among these 4 groups, the techniques are crucial for enhancing the quality of therapy. One critical CBT therapeutic technique strategically uses Socratic questioning [17]. On the basis of the pedagogical style of the ancient Greek philosopher Socrates, this method is a form of guided discovery that encourages clients to critically examine and articulate their thought patterns to facilitate cognitive change and distress reduction [18,19]. Socratic questioning helps clients deeply contemplate their problems and perspectives. Questions such as “Why do you think that way?,” “What evidence supports this perspective?,” and “Have you considered other perspectives?” can be adjusted to specific problems or situations. These flexible templates can be applied to various concerns and situations. The process involves carefully considering the content, timing, and sequence of questions adjusted to an individual’s cognitive processes and emotional state, emphasizing the quality of queries when engaging the client in Socratic questioning [2,4,20]. Therapists observe the content of clients’ utterances, changes in their voices, and facial expressions to understand changes in their mood [2,4].

Recent studies have started to investigate the application of Socratic questioning in automated CBT systems such as ECAs. Kimani et al [14] and Shidara et al [21] explored ways to implement Socratic questioning in CBT delivered by ECAs. The former work provided Socratic questions according to a dialogue context using a flowchart of selectable inputs and reduced the specific anxiety about giving a presentation. The latter study set up 2 dialogue scenarios, with and without Socratic questioning, and evaluated them in a comparative experiment. The group with the scenario containing Socratic questioning showed a larger reduction in negative moods. Both studies suggest that Socratic questioning by ECAs promotes cognitive changes and distress reduction.

However, there is a significant gap in current research. Existing studies have not explored how to automatically adapt Socratic questioning. Ideally, ECAs should adapt CBT strategies according to users’ psychological states, just like human therapists do. Specifically, there is limited research on this topic. We must focus on the adjustment of the number of questions or its types. Therefore, we set our goal to personalize CBT strategies based on an individual’s current psychological state.

This study addresses this gap by introducing an adaptive method to improve the effectiveness of CBT using ECAs. This method adjusts the number of questions during a therapy session based on the detected level of psychological distress. The main goal is to provide questions according to the user’s level of distress to improve the therapeutic interaction. To achieve this goal, we formulated the following research questions:

1. *Research question 1*: Can modifying the number of questions based on the user’s level of psychological distress lead to better therapeutic outcomes?

To address this question, our system uses a machine learning model with natural language processing techniques to estimate a user’s psychological state during a CBT session. Our system can automatically detect psychological distress, allowing it to adapt to the user’s mental state without using psychological scales such as the Kessler Psychological Distress Scale (K6) in each session. Furthermore, by integrating the distress detection model, our system can estimate the user’s distress in real time during the CBT session. This functionality is intended to improve the effectiveness of CBT with an ECA. In this sense, we present the hypothesis 1.

Hypothesis 1: In a condition with the adaptive number of questions, improvement in distress and emotion and cognitive change will be better than in a condition with a random number of questions.

2. *Research question 2*: Is there a clear benefit to modifying the number of questions to match the user’s specific psychological state rather than setting a fixed optimal number for all?

For this question, we emphasized the value of customization, recognizing that the most effective balance of questions may vary from user to user. Therefore, we aimed to clarify the therapeutic benefit of determining the distress reduction moments. Through this investigation, we aimed to identify the most effective strategy: changing the number of questions or setting a user-specific number of questions. Therefore, we propose hypothesis 2.

Hypothesis 2: The adapted question amount has better improvement than both the too few questions amount and the too many questions amount in terms of the users’ distress improvement, emotion improvement, and cognitive change.

To validate these hypotheses, we tested it experimentally by comparing 2 conditions: the adapted number of questions condition and the random number of questions condition. We assessed the reduction of psychological distress and negative emotional states and cognitive changes in both conditions. We also analyzed the influence of deviations in the number of questions from the model’s estimated value on the effectiveness of the ECAs.

The incorporation of a distress detection model into an ECA system is essential to our method. In the *Psychological Distress Detection and Conversational Scenario* section, we describe our psychological distress model and the design of the agent architecture.

### Psychological Distress Detection and Conversational Scenario

#### System Overview

**Figure 1:**
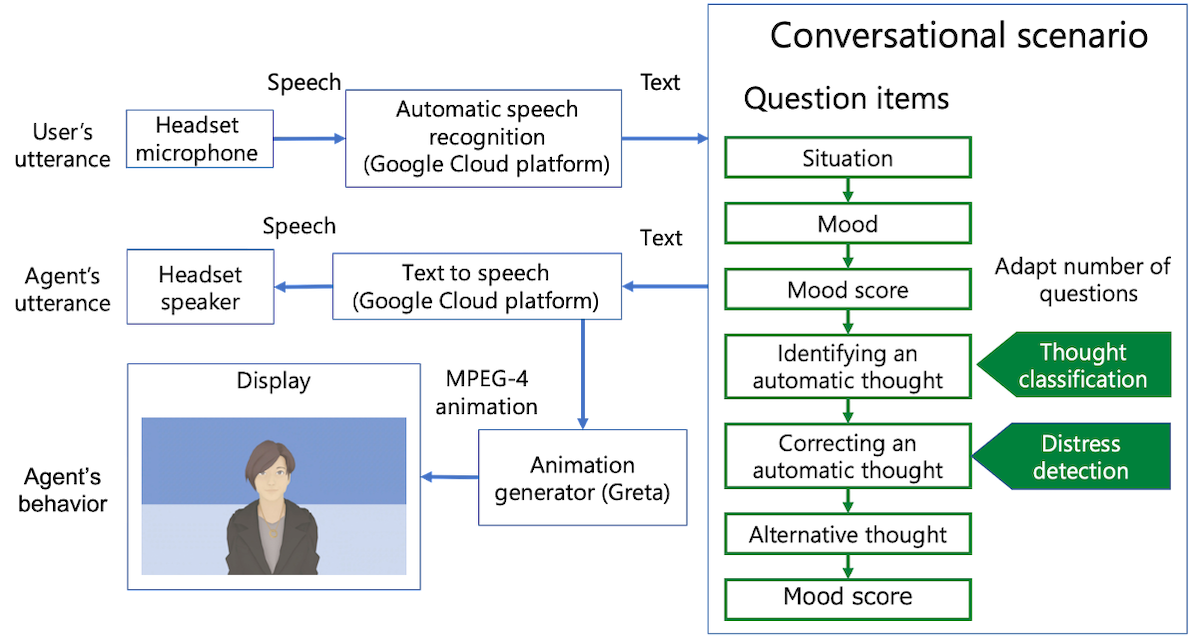
A framework of an embodied conversational agent and a conversational scenario for cognitive behavioral therapy. MPEG-4: Moving Picture Experts Group Phase 4.

**Figure 2:**
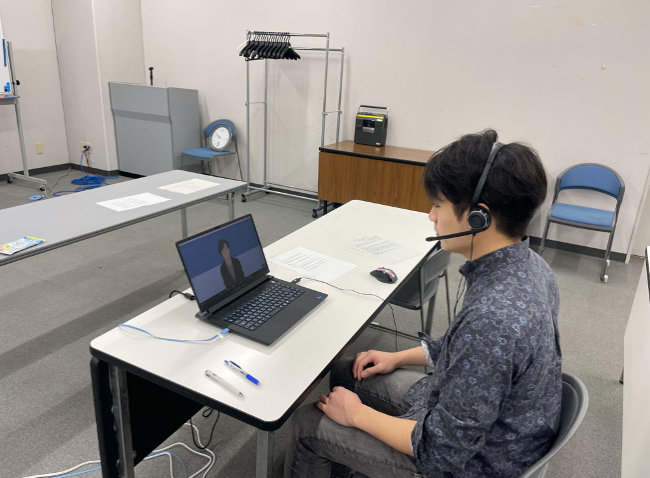
Conversation based on cognitive behavioral therapy between the user and the embodied conversational agent.

Figure 1 shows the overview of our system, including the abstract scenario. The detailed scenario can be found in Multimedia Appendix 1. This ECA uses an ECA toolkit called Greta (Institut des Systèmes Intelligents et de Robotique in Paris) [22]. Its animation’s appearance uses a version identified as more acceptable to Japanese people [23]. We used the ja-JP-Wavenet-B of Google’s Text-to-Speech service to generate the synthesized speech of a Japanese female voice. We used a laptop (i7, 32GB RAM, Dell Inc) for the ECA implementation and recorded the participants’ facial expressions and speech with its built-in camera. A microphone and a speaker were built into a headset (Sennheiser). Figure 2 shows a conversation between a user and our ECA.

In this study, we developed a question scenario based on the principles of cognitive restructuring [2,4], a key component of CBT. The scenario was supervised and reviewed by a psychiatrist to ensure clinical accuracy and relevance. The CBT process involves 3 main steps: identifying automatic thoughts, correcting them, and examining new balanced thoughts.

The first step in the scenario involves identifying automatic thoughts by describing the situation, the mood, and the automatic thoughts themselves. Typically, the situation and mood are described first, followed by identifying the automatic thoughts that occur between the situation and the mood. When describing mood, participants also report the intensity of their moods using a score, which is a percentage ranging from 0 (not stressed at all) to 100 (the most stressed they have ever felt). In this step, the users select their strongest automatic thought. Although multiple automatic thoughts may arise from a single situation, focusing on the strongest thought allows for the largest reduction of mood score.

In this study, we incorporated a classification model developed by Shidara et al [21] that determines whether automatic thoughts are expressed. Their model, which helps users accurately identify their automatic thoughts, was integrated into the response selection component of the ECA. When the ECA asks the user about his automatic thoughts, the model takes the response as input and determines whether the answer is a thought or something other than a thought, such as a mood or situation. If the response is deemed to be something other than a thought, the agent asks another question to elicit an automatic thought. If the answer is a thought, the agent proceeds to the thought-correction section. The model, constructed using Bidirectional Encoder Representations from Transformers [24], demonstrated a high estimation *F*_1_-score of 0.81.

Once the automatic thoughts have been identified, participants correct their thoughts by comparing them with the facts of the situation. The CBT system poses questions designed to increase participants’ awareness of overlooked facts and provides distance from their automatic thoughts to view them more objectively. After correcting their automatic thoughts, the participants were asked to state their new balanced thoughts. This step involved considering the insights gained while correcting automatic thoughts and generating new balanced thoughts. At the end of this process, participants reported their mood score again to confirm a reduction of their negative mood scores after deriving new balanced thoughts.

#### Adaptation of Number of Questions

In our developed scenario, multiple questions were included for correcting automatic thoughts. This step ensured that the standardized scenario effectively improved participants’ mood by incorporating multiple questions. In actual CBT sessions, questions are adjusted according to an individual patient’s condition and level of distress.

Our ECA posed questions aimed at helping participants notice overlooked facts and gain distance from their automatic thoughts to view them more objectively. In the developed scenario, multiple Socratic questions were included for correcting automatic thoughts. This step ensured that the standardized scenario effectively improved participants’ mood by incorporating multiple questions. The number of questions posed for correcting automatic thoughts was adapted based on the detected level of the participant’s psychological distress. After posing questions that correct an automatic thought, the participant’s responses were used to identify their current psychological distress state. If distress is detected, the system continues to pose additional questions that prompt the participants to correct their thinking from various perspectives. This process gently nudges participants to reconsider their automatic thoughts, offering them the opportunity to alter their perspective and reduce their distress. The system was designed using a pool of 21 Socratic questions to prompt the correction of automatic thoughts. If a participant continues to exhibit high levels of psychological distress, the agent can pose up to 20 additional questions to assist them in working through their distress.

If the estimated value of the detection model is nondistressed, the agent proceeds to the next step, where the participant composes a balanced alternative thought. This step was based on the insights gained in the previous step, which is the correction of automatic thoughts. After this process, the participant again reported a negative mood score, which was expected to confirm a less negative mood.

#### Pretraining of Detection Models to Address Japanese Conversation

In our quest to dynamically adapt the number of questions asked during a CBT session, psychological distress in users must be accurately detected. In this context, machine learning models have been developed to estimate psychological distress [25-29], often using publicly available data sets, such as the Distress Analysis Interview Corpus/Wizard-of-Oz (DAIC-WOZ) database [30]. Unfortunately, this data set primarily contains English language data focused on assessing depressive tendencies rather than providing mental health care. Therefore, our challenge is to adapt these models to suit a Japanese language context, especially for the CBT domain.

To address this challenge, we first applied a deep learning architecture of the depression detection model proposed by Li et al [31]. Their model identifies depressive tendencies in interactions with ECAs by classifying presence or absence into 2 categories. It combines recurrent neural network and long short-term memory networks and uses multitask learning for depression detection. Multitask learning enables simultaneous training on multiple tasks, leveraging shared knowledge to improve generalization, use relationships between tasks, and reduce overfitting. In the study by Li et al [31], the model achieved a high *F*_1_-score of 0.71.

**Figure 3:**
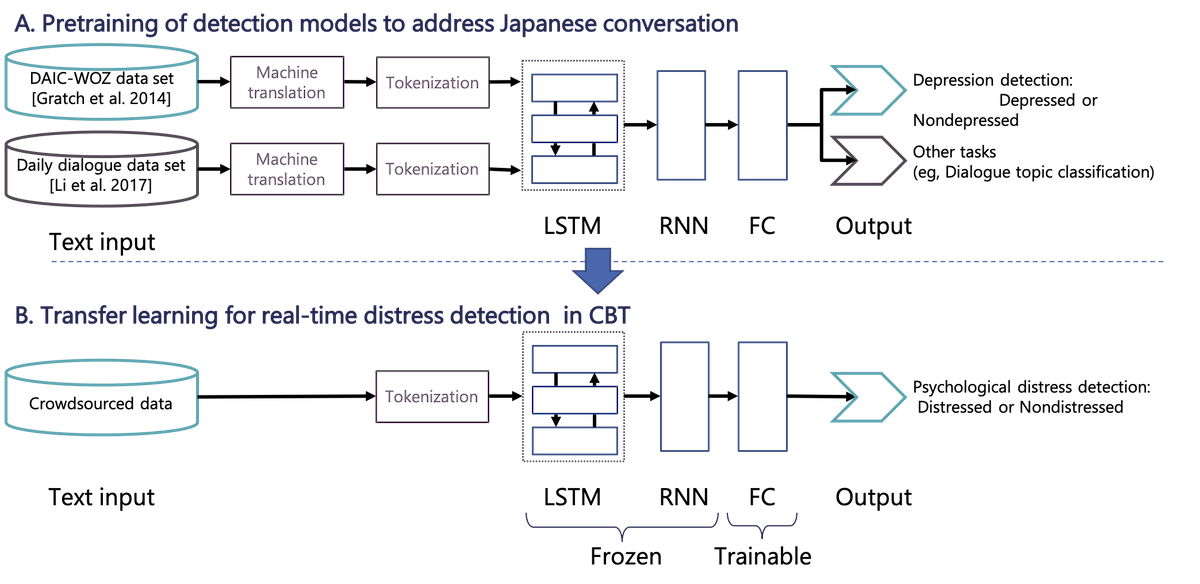
(A) Pretraining of detection models to address Japanese conversation data sets. (B) Transfer learning for real-time psychological distress detection in CBT. CBT: cognitive behavioral therapy; DAIC-WOZ: Distress Analysis Interview Corpus/Wizard-of-Oz; FC: fully connected; LSTM: long short-term memory; RNN: recurrent neural network.

Figure 3 [30,32] shows the training flow of the psychological distress detection model [30,32]. For the training process, we used a combination of pretraining and transfer learning. First, we translated the original training data in the study by Li et al [31] from English into Japanese using machine translation software and pretrained the model with reference to the settings in the study by Li et al [31]. We then constructed a Japanese CBT data set using crowdsourcing and conducted transfer learning on the detection model.

In the pretraining, we used data from 2 sources: the DAIC-WOZ data set [30] and the DailyDialog data set [32]. The DAIC-WOZ data set consists of 189 two-party English interviews between participants and Ellie, an animated ECA interviewer. Participants were evaluated using the Patient Health Questionnaire-8 scale [33]. Participants with a Patient Health Questionnaire-8 score ≥10 were considered depressed [33]. The DailyDialog data set [32] is an English dialogue collection containing 13,118 two-party daily life conversations. The data set included 3 expert-annotated information layers: 7 categorical emotions [34], 4 dialogue acts, and 10 dialogue topics. We maintained the original separation into training, validation, and test sets for both data sets. We used a transformer translation model, implemented with HuggingFace transformers [35], for the English-Japanese translation of both data sets.

We retained the original implementation details of the study by Li et al [31]. We trained the model for a maximum of 100 epochs with early stopping based on the macro *F*_1_ metric for depression classification. We used cross-entropy loss and a batch size of 1 for the DailyDialog and DAIC-WOZ. Tokenization was performed using the MeCab library [36], and we constructed word embeddings with a default dimension of 128. The turn-level encoder consisted of 1 hidden layer and 128 output neurons. We tuned the document-level recurrent neural network layers within a range of {1, 2, 3} and the hidden size within a range of {128, 256, 512}. The model parameters were optimized using the Adam optimizer [37] with a learning rate of 0.001. Dropout rates were set to 0.1 for both the turn and document encoders.

#### Transfer Learning for Real-Time Distress Detection in CBT

After pretraining the model with the translated data, we implemented transfer learning by replacing the fully connected layer. This transfer learning process allowed us to leverage the original model architecture while shaping it to our specific distress detection task in CBT. In this transfer learning phase, we incorporated only 3 types of user utterances corresponding to the situation, mood, and automatic thought responses within the CBT process. This approach was adopted to enable real-time distress detection during CBT sessions with the ECA. When the detection model makes inferences, it inputs the 3 most recent utterances. In this way, the distress detection model enables real-time detection in a CBT session.

Our crowdsourced data set was initially collected from 100 crowd workers from the general public. However, upon visual inspection of the data, it became apparent that the responses from some participants were not adequately informative or valid for our purpose. Therefore, we excluded data from 6 participants as likely outliers or nonresponsives. Thus, the final data set for our analysis consisted of responses from 94 participants. In the data collection, we assessed the level of psychological distress in our crowdsourced data set with the Japanese version of K6 [38] (Japanese version [39]). This scale is a concise and reliable self-report tool that assesses the level of psychological distress experienced by individuals in the previous 30 days. This 6-item scale measures the frequency of symptoms related to anxiety and depression, making it a valuable instrument for detecting and evaluating mental health disorders in both clinical and research settings. The items are rated on a 5-point Likert scale, from 0 (none of the time) to 4 (all of the time), leading to a total score range of 0 to 24. Higher scores indicate greater psychological distress. In this study, following Sakurai et al [40], a K6 score of ≥5 was considered distressed, and a score of <5 was considered nondistressed. We constructed a psychological distress detection model that classified these 2 classes. Of the 94 participants, 63 (67%) were categorized as distressed (K6 score of ≥5) and 31 (33%) as *nondistressed* (K6 score <5). We used stratified sampling to automatically split the data into training, validation, and testing subsets. We used 55% (52/94) of the participants for training, 19% (18/94) of the participants for validation, and 26% (24/94) of the participants for testing.

## Methods

### Ethical Considerations

The research ethics committee of the Nara Institute of Science and Technology reviewed and approved this study (2019-I-24-3). We engaged a human resources company to advertise and recruit participants. All participants provided written informed consent before participating in the experiment. The participants were compensated with an honorarium, which was paid by the human resources company. Study data were anonymized.

### Participants

This study was conducted in February and March 2023. Eligibility criteria for the experimental participants were as follows: (1) aged between 18 and 65 years, (2) having no hearing impairments, and (3) ability to speak Japanese. For the analysis of this research, 49 participants were allocated into 2 conditions to assess the impact of varying the number of Socratic questions based on detected levels of psychological distress during CBT sessions with an ECA.

In addition to these participants, a separate group (of 26 participants) was involved in a distinct experimental condition, designed for a different research focus. Although data for this separate group were collected concurrently with the main study, the data from this separate group are not relevant to the current analysis and have not been included in this paper. The objective of collecting the data from this separate group was to analyze whether an extreme number of questions were stressful and discouraged cognitive changes. It is expected that participants in this separate group might experience or perceive the system rather than personal emotional challenges such as depression or anxiety. Specifically, participants may be dissatisfied with how the system works or interacts rather than with their internal struggles. Although this system-related dissatisfaction is also important, it is beyond the scope of this study. Only the 2 conditions were used in this study to focus on the person’s nonadaptive thoughts and moods regarding their problems.

Table 1 shows the demographics and baseline characteristics of the participants who were recruited through an external participant recruitment service. We also included 1 participant with a history of a mental health issue in the adapted number of questions condition. This participant was currently in remission and not taking any medication. This participant was recruited through a company that provides employment transition services for people with a history of mental health issues.

**Table 1:** Participants’ demographics and baseline characteristics (N=49).

Characteristics			Adapted number of questions condition		Random number of questions condition
**Total number of participants, n (%)**			25 (100)		24 (100)
	Without mental health issues, n (%)	24 (96)		24 (100)	
	With a history of a mental health issue, n (%)	1 (4)		0 (0)	
**Sex, n (%)**					
	Male	12 (48)		13 (54)	
	Female	13 (52)		11 (46)	
Age (years), mean (SD)			41.72 (12.79)		38.88 (14.89)
K6^a^, mean (SD)			4.08 (4.31)		4.38 (3.10)

^a^K6: Kessler Psychological Distress Scale.

To confirm whether there was any bias in the age of the participants and the preexperiment K6 scores between the 2 conditions, we conducted a 2-sided Welch *t* test and calculated Cohen *d* values. As a result, for age, Cohen *d* was 0.21 and the *P* value was .48; for preexperiment K6 scores, Cohen *d* was −0.08 and the *P* value was .78. These results indicate that there was no significant bias in age and preexperiment K6 scores between the 2 conditions.

### Experimental Conditions

**Figure 4:**
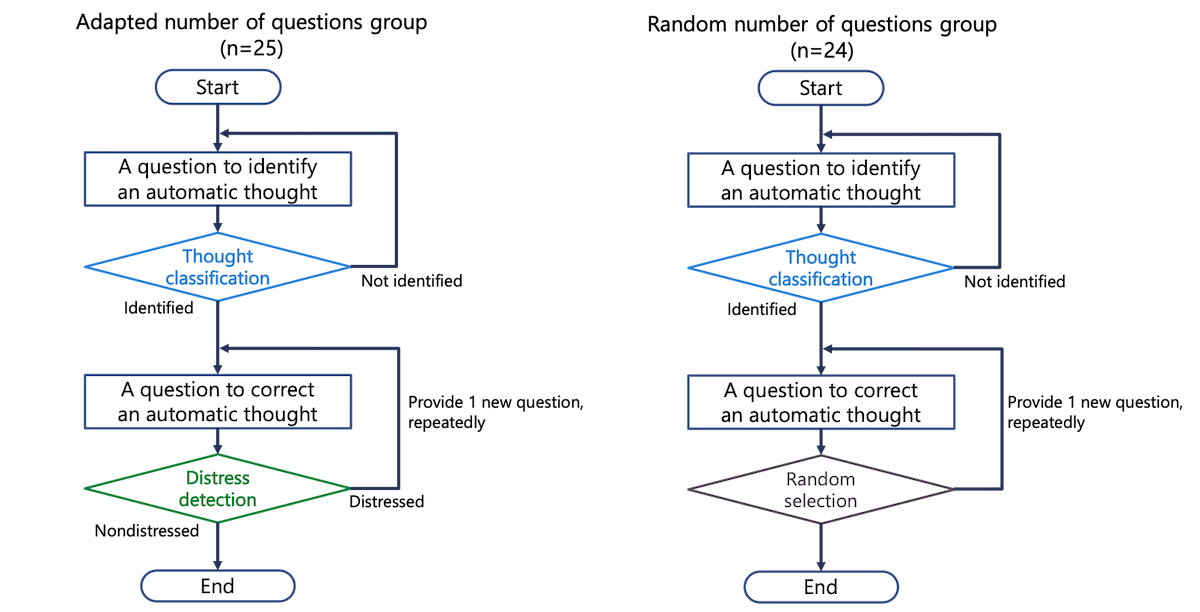
Diagram of the experimental conditions. The adapted number of questions condition, which includes the psychological distress detection model, versus the random number of questions condition, which does not include the distress detection model.

Figure 4 illustrates the conversation scenarios for our 2 experimental conditions: the adapted number of questions and the random number of questions. In the adapted number of questions condition, the ECA used a distress detection model. This model continuously estimated the participant’s level of distress after each answer for a thought-correction question. If the model detects distress, it prompts the agent to ask a new question to correct the automatic thought. This process is repeated until the model detects no distress or the maximum number of questions is reached. In contrast, the condition of a random number of questions does not use the distress detection model. Instead, the system randomly determines the number of questions without considering the participant’s distress state. In both conditions, the number of questions asked to correct automatic thoughts varied from a minimum of 1 to a maximum of 21.

The condition assignment was not concealed from the experimental examiner. Participants were automatically assigned to conditions based on their age, sex, and K6 scores (a self-reported psychological distress scale). The day before the experiment, the participants responded to these 3 items using a questionnaire administered through Google Forms. On the basis of the questionnaire results, the conditions were divided such that no imbalance existed between the conditions in terms of K6, age, and sex. The participants conducted the experiment according to their predetermined condition assignments. They were unaware of their condition assignment and were not informed of the differences between the conditions.

Before the experiment, an experimenter explained this to the participants who signed the consent forms. They then completed the K6 questionnaire and read a publicly available leaflet explaining CBT [41]. The experiment consisted of the same questions as those in the ECA dialogue scenario created in our previous study [21]. The experiment was completed within 1 hour, including its briefing and preparation.

### Measures

#### Psychological Distress

K6 was used as the rating scale to assess the psychological distress of our participants. Before the experiment, we obtained the K6 results for the previous 30 days. After the experiment, the participants were asked if any of the K6 items had changed after the CBT. The postexperimental measures differed from the original protocol, and we asked about the changes solely for analyzing our experiment. We calculated the change in psychological distress using equation 1.


Change in psychological distress = K6 score (pre) − K6 score (post) **(1)**

Before the session, we measured the Quick Inventory of Depressive Symptomatology [42] (Japanese version [43]), which assesses depressive tendencies.

#### Cognitive Change

The Cognitive Change-Immediate scale [44] is a 5-item self-report measure that assesses the extent of the cognitive changes experienced by users during a single CBT session within therapy sessions. Items were rated on a scale from 0 (not at all) to 6 (completely), yielding a total score ranging from 0 to 30. Higher scores indicate a greater degree of cognitive change experienced by the participants.

#### Mood Change

A mood score is a numerical representation of the intensity of self-reported negative emotions experienced by the participants. The scores ranged from 0 to 100, with higher scores indicating more severe distress. Participants verbally provided their mood scores at 2 time points during scenarios involving CBT sessions with the ECA. The initial mood score was used for prethought correction, and the subsequent score was used for postthought correction. Specifically, participants were asked, “On a scale of 0 to 100, where 0 is no problem at all and 100 is a huge problem, how intense is that feeling?” These questions are commonly used to assess relief from negative moods [2,4]. Participants provided their responses verbally during the CBT. The participants’ moods were characterized using such language as anxiety, depression, sadness, feelings of inferiority, and fatigue. In this study, these negative emotional states were collectively assessed under an umbrella term, negative mood, which focuses exclusively on mood scores to evaluate the changes in these states. This approach allowed us to assess *mood change*. Following a previous methodology [45], mood change was calculated using equation 2.


Mood change = [Mood score (pre) − Mood score (post)]/Mood score (pre) **(2)**

This calculation is a measure of the degree of change in a participant’s negative mood, where a larger positive value indicates a decrease in negative mood.

#### State–Trait Anxiety

The State–Trait Anxiety Inventory [46] (Japanese version [47]) is a validated, self-report questionnaire that assesses both state anxiety (temporary and situational anxiety) and trait anxiety (general and stable anxiety) in adults. The State–Trait Anxiety Inventory consists of two 20-item scales: the State–Trait Anxiety Inventory-State (STAI-S) and the State–Trait Anxiety Inventory-Trait (STAI-T). The STAI-S measures the respondent’s current anxiety levels and feelings of apprehension, tension, and nervousness. It assesses the extent to which an individual is experiencing anxiety in response to a specific situation or event. Items are scored from 1 (not at all) to 4 (very much), where higher scores indicate more severe state anxiety. The STAI-T evaluates a respondent’s general tendency to experience anxiety as a stable personality characteristic. It focuses on the feelings of anxiety that are not associated with a particular event or situation; instead, it reflects an individual’s overall tendency to become anxious. Items are scored from 1 (almost never) to 4 (almost always), where higher scores represent greater trait anxiety levels. Both STAI-S and STAI-T scores can range from 20 to 80, and higher scores denote greater anxiety. We calculated the change in STAI-S using equation 3.


Change in STAI-S = STAI-S (pre) − STAI-S (post) **(3)**

This calculation is a measure of the degree of change in a participant’s state anxiety, where a larger positive value indicates a decrease in state anxiety.

### Statistical Analysis

We conducted an investigation to address hypothesis 1. This hypothesis states that an adaptive selection of the number of questions based on detected psychological distress would have superior effects in improving mental states. This approach was compared with the one in which the number of questions was randomized. For this investigation, we focused on several metrics, including K6 scores, mood scores, STAI-S, and cognitive change. Both within-condition and between-condition comparisons were conducted within these metrics. Owing to the inability to confirm the normal distribution of these measures in both the adapted number of questions condition and the random number of questions condition, we chose to use nonparametric tests for our analyses.

For within-condition comparisons, we assessed the differences in scores before and after the CBT session for each measurement in both conditions. The Wilcoxon signed-rank test was used to analyze these within-condition differences, illustrating the effect of the session on the adapted and random number of questions conditions individually.

For between-condition comparisons, we compared the pre- and postsession scores for each measurement between the adapted and random number of questions conditions. The Mann-Whitney *U* test was used for this analysis. In addition, we conducted a comprehensive analysis across 4 key parameters: change in psychological distress, cognitive change, mood change, and change in the STAI-S. This analysis involved multiple 1-sided Mann-Whitney *U* tests under the assumption that the adapted number of questions condition would show higher effects on each measure than the random number of questions condition.

We conducted a subgroup analysis to examine hypothesis 2, which asserts that adapting the number of questions according to the participant’s psychological distress is more effective. We categorized the participants into 3 subgroups based on the difference between the number of questions asked and the distress detection model’s estimation: the *same group* (participants who were asked the same number of questions as determined by the distress detection model), the *fewer group* (fewer questions were asked than determined by the distress detection model), and the *more group* (more questions were asked than determined by the distress detection model). In this analysis, we combined participants from both the adapted number of questions condition and the random number of questions condition. We did this because we expected too few participants in the same group within the random number of questions condition. We compared the same group with the fewer and more groups, expecting superior outcomes for the same group. This comparison used a 1-sided Mann-Whitney *U* test to compare the cognitive change, mood change, change in K6 scores, and change in the STAI-S across the subgroups.

In addition, we conducted further analysis to validate hypothesis 2. In this step, we examined the differences in various measurements across specific question counts. We focused on groups divided by question amount, such as 10, in the condition of a random number of questions. These were then compared with the participants in the adapted number of questions condition. Owing to the limited sample size in each category, we restricted our examination to qualitative analysis. In this comparative analysis, we did not conduct statistical testing. Furthermore, we reported the number of nondistressed and distressed values automatically detected for each individual question. These analyses aimed to demonstrate the effectiveness of a flexible approach to the number of questions presented.

Although we collected usability questionnaires and subjective evaluation feedback from the participants, this information was not included in the primary analysis of this study.

## Results

### Model Construction Results

Pretraining our detection models to address Japanese conversation yielded the following results: for the English data set (original condition and reproductive experiment), the accuracy was 0.72, precision was 0.67, recall was 0.58, and *F*_1_-score was 0.62. When the data set was translated by an automatic translation model into Japanese, the models achieved an accuracy of 0.64, precision of 0.59, recall of 0.60, and an *F*_1_-score of 0.59, demonstrating that we maintained equivalent performance levels despite the change in language.

We further applied transfer learning to the pretrained model with Japanese conversation for real-time distress detection in CBT. The results of implementing this transfer learning are as follows: In the condition where only pretraining was applied, the model achieved an average accuracy of 0.55, precision of 0.47, recall of 0.48, and an *F*_1_-score of 0.48 across 5 evaluation trials. When both pretraining and transfer learning were implemented, the average scores improved, with the model achieving an accuracy of 0.70, precision of 0.60, recall of 0.63, and an *F*_1_-score of 0.61. The model that demonstrated the highest performance during these trials, exhibiting an accuracy of 0.75, recall of 0.69, precision of 0.72, and an *F*_1_-score of 0.70, was selected for integration into the conversational scenario.

### Experimental Results

Table 2 presents the pre- and postsession scores for both the adapted and random number of questions conditions across various metrics.

**Table 2:** Pre- and postsession measures for the adapted and random number of questions conditions.

			Adapted number of questions condition (n=25)		Random number of questions condition (n=24)		Between-condition comparison		
							Cliff delta		*P* value
**K6^a^**									
	Presession measures, mean (SD)	4.08 (4.31)		4.38 (3.10)		−0.07		.40	
	Postsession measures, mean (SD)	3.04 (4.19)		4.30 (3.17)		−0.17		.04	
	Within-condition comparison, effect size *r* (*P* value)	−0.49 (.01)		−0.09 (.65)		—^b^		—	
**Mood score**									
	Presession measures, mean (SD)	57.44 (22.30)		65.63 (20.76)		−0.10		.23	
	Postsession measures, mean (SD)	46.12 (24.53)		57.92 (17.38)		−0.11		.19	
	Within-condition comparison, effect size *r* (*P* value)	−0.66 (.001)		−0.44 (.03)		—		—	
**STAI-S^c^**									
	Presession measures, mean (SD)	41.40 (12.43)		40.75 (9.53)		−0.01		.89	
	Postsession measures, mean (SD)	38.40 (10.46)		38.21 (10.27)		0.02		.85	
	Within-condition comparison, effect size *r,* (*P* value)	−0.29 (.14)		−0.39 (.05)		—		—	
**QIDS^d^**									
	Presession measures, mean (SD)	5.00 (4.62)		4.83 (3.67)		−0.03		.74	
**STAI-T^e^**									
	Presession measures, mean (SD)	44.10 (12.5)		42.1 (10.8)		0.04		.65	
Time spent (second), mean (SD)			494.96 (161.10)		661.40 (289.20)		−0.10		.26
Number of questions, mean (SD)			4.76 (4.48)		8.83 (6.52)		−0.17		.03

^a^K6: Kessler Psychological Distress Scale.

^b^Not available.

^c^STAI-S: State–Trait Anxiety Inventory-State.

^d^QIDS: Quick Inventory of Depressive Symptomatology.

^e^STAI-T: State–Trait Anxiety Inventory-Trait.

The average time spent on the dialogues in the adapted number of questions condition was 494.96 (SD 161.10) seconds, whereas the random number of questions condition spent an average of 661.40 (SD 289.20) seconds. However, it should be noted that owing to system issues, we were unable to record the time spent by one participant in each condition. As a result, the reported mean and SD for time spent exclude the data from these 2 participants. All other measurements were successfully recorded for these participants.

The average number of questions asked to correct an automatic thought was 4.76 (SD 4.48) for the adapted number of questions condition and 8.83 (SD 6.52) for the random number of questions condition.

We evaluated the distress detection model’s performance in the adapted number of questions condition. Specifically, we evaluated its accuracy in correctly identifying the nondistressed states, indicating when to stop asking Socratic questions. The model’s performance was measured by comparing its output (0 denoting nondistressed) with the participants’ actual postexperiment psychological distress states. The model correctly identified nondistressed state in 72% (18/25) of the instances.

In the presession phase, the K6 scores did not significantly differ between the 2 conditions. However, a significant difference was observed in the postsession phase; the adapted number of questions condition recorded a notably lower K6 score, indicating a substantial reduction in distress compared with the random number of questions condition. On other measures, the pre- and postsession scores were not significantly different between the 2 conditions.

A closer look at the within-condition comparisons revealed that the adapted number of questions condition experienced a decrease in scores across several metrics following the session, albeit to varying degrees. The adapted number of questions condition showed more marked improvements, especially in the K6 and mood scores, with significant reductions after the session, as evidenced by Cliff delta values of −0.49 (*P*=.01) and −0.66 (*P*=.001), respectively. In contrast, although the random number of questions condition also showed a reduction in the mood score, characterized by a medium effect size (Cliff delta=−0.44; *P*=.03), the change in the K6 score was negligible between the pre- and postsession periods.

Table 3 presents the comparative results of changes in each measurement. We conducted a 1-sided Mann-Whitney *U* test to compare the cognitive change, mood change, change in the K6 scores, and change in the STAI-S between the adapted number of questions condition and the random number of questions condition. The results indicated that there was no significant difference in the change in psychological distress, mood change, and change in STAI-S between the 2 conditions, with *P* values of .11, .16, and .54, respectively. However, for cognitive change, a relatively large effect size was observed with a Cliff delta of 0.12, and the *P* value was .07, showing a trend toward significance, suggesting that the adapted number of questions condition exhibited a tendency for greater cognitive change compared with the random number of questions condition.

**Table 3:** Comparative results of changes in each measurement using nonparametric analysis of 1-sided Mann-Whitney *U* test for differences between conditions.

	Adapted number of questions condition, mean (SD)	Random number of questions condition, mean (SD)	Cliff delta	*P* value
Change in psychological distress	1.04 (1.95)	0.08 (1.28)	0.09	.11
Cognitive change	16.3 (6.38)	13.7 (5.59)	0.12	.07
Mood change	0.22 (0.31)	−0.05 (0.88)	0.08	.16
Change in STAI-S^a^	3.00 (8.10)	2.54 (5.99)	−0.009	.54

^a^STAI-S: State–Trait Anxiety Inventory-State.

To investigate hypothesis 2, we conducted a subgroup analysis as described in the *Statistical Analysis* subsection. We categorized the 49 participants from the adapted and random number of questions conditions based on the deviation between the differences in the questions asked and the estimated value of the model. Of the 21 participants, 7 (14%) received fewer questions than estimated and 14 (29%) received more questions than estimated, and for 28 (57%) participants, the number of questions matched the estimated value, denoted as the same group.

Table 4 shows the mean and SD for each subgroup. For cognitive changes, comparisons between the same and fewer groups showed a small effect size (Cliff delta=0.24; *P*=.03), indicating a significant difference. In contrast, comparisons between the same and more groups showed a negligible effect size (Cliff delta=−0.04; *P*=.65), indicating no significant difference. For the change in psychological distress, the comparisons between the same and fewer groups showed a negligible effect size (Cliff delta=0.03; *P*=.40), indicating no significant difference. Similarly, comparisons between the same and more groups showed a negligible effect size (Cliff delta=0.04; *P*=.34), indicating no significant difference. For mood changes, the comparisons between the same and fewer groups showed a negligible effect size (Cliff delta=0.08; *P*=.26), indicating no significant difference. Similarly, the comparisons between the same and more groups showed a negligible effect size (Cliff delta=0.01; *P*=.44), indicating no significant difference. Finally, for changes in the STAI-S, comparisons between the same and fewer groups showed a negligible effect size (Cliff delta=−0.03; *P*=.46), indicating no significant difference. The comparisons between the same and more groups also showed a negligible effect size (Cliff delta=−0.07; *P*=.77), indicating no significant difference.

**Table 4:** Mean and SD in the same, more, and fewer groups.

	Same, mean (SD)	Fewer, mean (SD)	More, mean (SD)
Change in psychological distress	0.86 (1.93)	−0.14 (1.78)	0.36 (1.01)
Cognitive change	15.57 (6.51)	10.71 (4.79)	16.07 (5.14)
Mood change	0.20 (0.30)	−0.45 (1.57)	0.13 (0.32)
Change in STAI-S^a^	2.53 (7.81)	1.86 (6.04)	3.71 (6.32)

^a^STAI-S: State–Trait Anxiety Inventory-State.

Table 5 illustrates the mean changes in various measurements such as distress, cognitive change, and mood change across different numbers of questions. As indicated in Table 4, the mean changes for the adapted number of questions condition were 1.04 for change in psychological distress, 16.3 for cognitive change, 0.22 for mood change, and 3.00 for change in STAI-S. As indicated in Table 4, the mean changes for the adapted number of questions condition were 1.04 for change in psychological distress, 16.3 for cognitive change, 0.22 for mood change, and 3.00 for change in STAI-S. The bar graphs of these data can be found in Multimedia Appendix 2. For the random number of questions condition, it was observed that in most instances, the mean change was lower compared with the overall average of the adapted number of questions condition. However, a notable exception was observed at a question count close to the average question count for the adapted number of questions condition (mean 4.76, SD 4.48), specifically at 5 questions. At this count, the random number of questions condition exhibited slightly higher scores in both distress change and cognitive change compared with the adapted number of questions condition. Furthermore, a trend was observed in cognitive change, where an increase in the number of questions correlated with a larger change, indicating a potential area for further exploration and validation in subsequent studies. Despite these observations, it was consistently found that the adapted number of questions condition manifested superior effectiveness across various numbers of questions, indicating robustness in its application regardless of the question count.

**Table 5:** Mean change in each questionnaire across different numbers of questions posed to participants in the random number of questions condition. Instances where the mean is “not available” indicate that the number of participants (n) is 0. Instances where the standard SD is “not available” are due to the number of participants (n) being either 0 or 1.

Number of questions	Change in distress, mean (SD)	Mood change, mean (SD)	Cognitive change, mean (SD)	Change in STAI-S^a^, mean (SD)
1	0.67 (0.58)	0.21 (0.08)	12.67 (2.08)	1.33 (4.51)
2	−1.00 (1.41)	−0.03 (0.04)	4.50 (0.71)	4.00 (5.66)
3	0.50 (0.71)	0.08 (0.12)	11.00 (9.90)	4.00 (0.00)
4	0.00 (—^b^)	0.00 (—)	12.00 (—)	−5.00 (—)
5	1.50 (2.12)	0.10 (0.14)	18.00 (2.83)	1.50 (9.19)
6	0.50 (0.71)	0.13 (0.19)	7.00 (8.49)	5.00 (8.49)
7	—	—	—	—
8	0.00 (—)	0.17 (—)	13.00 (—)	1.00 (—)
9	−4.00 (—)	−4.00 (—)	11.00 (—)	−9.00 (—)
10	—	—	—	—
11	1.00 (—)	0.83 (—)	19.00 (—)	5.00 (—)
12	0.50 (0.71)	0.20 (0.00)	19.50 (3.54)	3.50 (3.54)
13	—	—	—	—
14	−1.00 (0.00)	−0.33 (0.47)	16.50 (0.71)	−0.50 (6.36)
15	—	—	—	—
16	—	—	—	—
17	0.00 (—)	0.00 (—)	15.00 (—)	−1.00 (—)
18	0.50 (0.71)	0.21 (0.06)	15.50 (4.95)	12.50 (9.19)
19	0.00 (—)	0.00 (—)	17.00 (—)	4.00 (—)
20	—	—	—	—
21	0.00 (—)	0.43 (—)	20.00 (—)	2.00 (—)

^a^STAI-S: State–Trait Anxiety Inventory-State.

^b^Not available.

Table 6 displays the number of detected values classified as nondistressed and distressed for each question aimed at correcting automatic thoughts. The bar graphs of these data can be found in Multimedia Appendix 3. In the adaptive question amount condition, questioning concluded with the detection of a participant’s first nondistressed value. Conversely, the process continued regardless of the values detected in the random question amount condition. Consequently, the total number of participants decreased as the number of questions increased. A significant proportion of participants were identified with nondistressed values, particularly within the initial 4 questions. However, as the number of questions reached a certain amount, the frequency of nondistressed value detection decreased, with none identified from questions 16 to 21. Meanwhile, distressed value detection persisted, with at least 1 individual exhibiting distressed values through the 21st question.

**Table 6:** Number of nondistressed and distressed values detected for each question aimed at correcting automatic thoughts, including only those participants who were presented with the same or a greater number of questions.

Number of questions	Number of destressed values	Number of nondestressed values
1	40	8
2	30	12
3	25	11
4	16	14
5	19	5
6	17	2
7	12	4
8	10	5
9	10	4
10	11	1
11	10	2
12	7	4
13	7	1
14	6	2
15	5	1
16	5	0
17	5	0
18	4	0
19	2	0
20	1	0
21	1	0

## Discussion

### Principal Findings

The primary aim of this study was to evaluate a method that adapts the number of questions in a CBT session based on the user’s level of distress, aiming to enhance the session’s effectiveness.

Hypothesis 1 posited that an adaptive approach to the number of questions selection, based on detected psychological distress, would lead to superior cognitive change and reduce psychological distress as well as negative emotional states compared with the random number of questions condition. The results partially supported this hypothesis. We observed a reduction in psychological distress from before the session to after the session in the adapted number of questions condition, whereas no such reduction was evident in the random number of questions condition. Furthermore, the level of distress after the session was significantly lower in the adapted number of questions condition than in the random number of questions condition. In contrast, no significant difference was observed in the changes in each measurement between the 2 conditions. These findings overall indicate that our approach contributed to optimizing the health care process and its outcomes.

A plausible reason for the significant improvement observed only in distress is that the deep learning model incorporated in this study was designed to detect the presence or absence of psychological distress. This suggests that although the CBT system’s adjustment of the number of questions was adjusted for distress, it was not necessarily adjusted for other measures. In cognitive models, cognition, including automatic thoughts, and reactions such as distress are considered distinct entities [2]. Relevant studies [19,44,45] explore the ways in which cognitive shifts can influence stress levels. Moving forward, it might be necessary to analyze the content of CBT and propose methods that enhance both cognitive and distress changes.

Hypothesis 2 proposed that better improvement would occur when the number of questions corresponded with the model’s estimated value. The data partially supported this, indicating more significant cognitive changes when the number of questions was aligned with the model’s estimate compared with when fewer questions were asked. This insight suggests the importance of precision in the number of questions selected to enhance the effectiveness of CBT delivered by ECAs. It was also implied that a large number of questions does not necessarily work effectively. Table 6 (Multimedia Appendix 3) shows that none of the participants were detected as nondistressed from questions 16 to 21. This result suggests that further questioning might not lead to significant improvements when there is no improvement in distress after a certain number of questions. Therefore, it would be prudent to set an upper limit on the number of questions that the system can ask.

We personalized the therapeutic process by ensuring a timely adjustment of the number of questions when the distress alleviation was projected to be insufficient. Our experimental data suggest that this approach significantly bolsters the effectiveness of ECAs in integrating thought-correction techniques into the therapy process. The data also revealed that maintaining a balance in the number of questions was critical for improving CBT’s effectiveness. Our findings highlight the potential of dynamic and personalized strategies in enhancing mental health care.

### Comparison With Prior Work

The field of mental health care has significantly evolved owing to advancements in artificial intelligence. Among these advancements, using ECAs to deliver mental health care services is particularly promising. Influential studies in this domain, including the work of Fitzpatrick et al [10], DeVault et al [48], and Fulmer et al [49], investigate the effectiveness of these text-based conversational agents or ECAs in mental health care. Furthermore, research by Inkster et al [11], Ghandeharioun et al [50], and Murali et al [51] has revealed the importance of these agents' ability to convey empathy.

These empathetic elements are crucial for creating a therapeutic alliance and supportive environment. However, they have not tapped into the CBT’s full potential to foster self-insight and bring about cognitive and behavioral changes. The transformative process in CBT requires a strategic use of questions. These questions aim to probe, challenge, and reshape negative thoughts and behaviors. This aspect of questioning remains underexplored in existing research.

In addition, research endeavors by Kimani et al [14] and Shidara et al [21] initiated the exploration of the application of Socratic questioning in CBT delivered by ECAs. These studies have shown promising results in fostering cognitive changes and reducing distress. However, they have not explored the dynamic adjustment of Socratic questioning based on users’ psychological states. This study aims to address this gap.

The integration of such adaptive strategies in mental health care, as demonstrated by our study, underscores the substantial contribution of our research in advancing the field. Our study introduces a novel component that modulates the number of questions according to the degree of the user’s psychological distress. The results of our study indicate that adapting to a user’s psychological distress by modulating the number of questions significantly reduced psychological distress compared with a random number of questions. This finding is in alignment with psychiatric insights suggesting that the modification of automatic thoughts through questioning may not yield sufficient effectiveness if such thoughts remain superficial [2].

### Limitations

When interpreting the results of this study, several limitations must be addressed. First, the cross-sectional design used in our research did not capture the long-term effects of ECAs on psychological distress. A long-term experiment is important to gain a deeper understanding of the lasting effects of ECAs and their potential role in improving mental health outcomes. Another limitation of our study is the small number of participants who exhibited high depressive tendencies. To enhance the generalizability of our findings, future research should involve a more diverse sample of participants, covering a broad range of psychological distress levels. This sample should include individuals with varying degrees of depression and other mental health concerns, ensuring that the results are more widely applicable to different populations that are experiencing various psychological issues. In addition, further research is needed on the effective selection of the number of Socratic questions to maximize the effectiveness of CBT. Techniques such as the WOZ method, in which a therapist operates an ECA, might shed light on this aspect [52].

### Conclusions

Our study provides evidence that adjusting the selection of questions based on an individual’s distress levels can significantly enhance CBT effectiveness. This adjusted approach allows for a more personalized health care, which can improve the therapeutic outcomes for individuals who are struggling with mental health issues, including anxiety and depression.

Our research highlights the importance of timely and appropriate reactions when an individual’s distress levels improve during therapy. By carefully monitoring and responding to these changes in distress, the ECAs can better support users’ progress. Overall, our study highlights the value of personalized and adaptive approaches in CBT, paving the way for more effective and responsive mental health care.

## Appendix

Multimedia Appendix 1 Conversational scenario of an embodied conversational agent.

Multimedia Appendix 2 Mean change across different numbers of questions posed to participants in the random number of questions condition. The blue bars represent the variation in mean change for measurements: (A) change in distress, (B) mood change, (C) cognitive change, and (D) change in State–Trait Anxiety Inventory-State (STAI-S). The green dashed line serves as a reference for comparison, indicating the average change in the adapted number of questions condition.

Multimedia Appendix 3 Number of detected values for each question aimed at correcting automatic thoughts: (A) nondistressed and (B) distressed values, including only those participants who were presented with the same or a greater number of questions.

## Abbreviations

CBT: cognitive behavioral therapy
ECA: embodied conversational agent
DAIC-WOZ: Distress Analysis Interview Corpus/Wizard-of-Oz
K6: Kessler Psychological Distress Scale
STAI-S: State–Trait Anxiety Inventory-State
STAI-T: State–Trait Anxiety Inventory-Trait

## Data Availability

The data sets generated during and analyzed during this study are not publicly available because of privacy and personal information protection concerns, but they are available from the corresponding author on reasonable request.
