# Supplementary material for: Adapting the Number of Questions Based on Detected Psychological Distress for Cognitive Behavioral Therapy With an Embodied Conversational Agent: Comparative Study
Source: JMIR Form Res. 2024 Mar 14;8:e50056. doi: 10.2196/50056 (PMC10979340; doi:10.2196/50056)
Supplement: Multimedia Appendix 1 [file formative_v8i1e50056_app1.docx]

Conversational scenario of virtual agent.

| No | Item | Utterance |
| --- | --- | --- |
| 1 | Greeting | Hello, my name is Rei, and I’m a therapist. Please introduce yourself. |
| 2 | Situation | Let's address your concerns together. Are you currently facing any issues or difficulties? If you feel comfortable doing so, please share what's troubling you. |
| 3 | Mood | How did you feel at that time? |
| 4 | Mood score | On a scale of 0 to 100, where 0 is no problem at all and 100 is a huge problem, how intense is that feeling? |
| 5 | Identifying an automatic thought 1 | What thoughts came to your mind at that time? |
| 6 | Identifying an automatic thought 2 | I see. Are there any others? Please share anything that comes to mind, and when you're finished, say, "That's all." |
| 7 | Identifying an automatic thought 3 | Please tell me the strongest thought among those that came to your mind. |
| 8 | Identifying an automatic thought 4 | Were you imagining something that might happen in the future or thinking about something that's already happening? Please provide an example of a thought that occurred to you. |
| 9 | Identifying an automatic thought 5 | What did you think about yourself in that situation? |
| 10 | Identifying an automatic thought 6 | What did you think about others in that situation? |
| 11 | Identifying an automatic thought 7 | What did you think would happen to you in the future? |
| 12 | Identifying an automatic thought 8 | How do you think others might feel about you in that situation? |
| 13 | Identifying an automatic thought 9 | Do you have any thoughts about other people or your environment? |
| 14 | Correcting an automatic thought 1 | I see. Those are called automatic thoughts. If they are correct, what is the basis for them? |
| 15 | Correcting an automatic thought 2 | On the other hand, if your automatic thoughts are incorrect, what evidence can you think of? |
| 16 | Correcting an automatic thought 3 | Can you think of any other perspectives regarding the situation that's troubling you, aside from your current automatic thoughts? |
| 17 | Correcting an automatic thought 4 | What would be the worst possible outcome in this situation? |
| 18 | Correcting an automatic thought 5 | If the worst possible outcome were to occur, how would you cope with it? |
| 19 | Correcting an automatic thought 6 | What would be the best possible outcome in this situation? |
| 20 | Correcting an automatic thought 7 | What do you think is the most realistic outcome in this situation? |
| 21 | Correcting an automatic thought 8 | What effect does believing in your current automatic thoughts have on you? |
| 22 | Correcting an automatic thought 9 | What effect would revising your current automatic thoughts have on you? |
| 23 | Correcting an automatic thought 10 | What would you say to a close friend if they were facing the same situation? |
| 24 | Correcting an automatic thought 11 | What actions could you take in response to your automatic thoughts? |
| 25 | Correcting an automatic thought 12 | What specifically complicates resolving your current troubling situation? |
| 26 | Correcting an automatic thought 13 | What would need to happen for you to feel like you have overcome this difficult situation? |
| 27 | Correcting an automatic thought 14 | Have you ever experienced a similar situation but didn't feel down? If so, what can you learn from that experience? |
| 28 | Correcting an automatic thought 15 | What constructive steps can you take in this situation? |
| 29 | Correcting an automatic thought 16 | What have you done so far to address this situation? |
| 30 | Correcting an automatic thought 17 | What would you do if the same situation were to happen again in the future? |
| 31 | Correcting an automatic thought 18 | Can you seek help from others? If so, from whom, and what kind? |
| 32 | Correcting an automatic thought 19 | What would your family or close friends say if they knew about your automatic thoughts? |
| 33 | Correcting an automatic thought 20 | Are there any strengths or positive qualities you might be overlooking in yourself? How could they help in this situation? |
| 34 | Correcting an automatic thought 21 | Are you blaming yourself for things that are beyond your control? What facts surface that could help you be more lenient towards yourself and your responsibilities? |
| 35 | Alternative/balanced thought | Perhaps there are other thoughts besides your automatic thoughts. If you come up with any, connect them to your automatic thoughts using "However". |
| 36 | Mood score | Now, how intense is the feeling you initially had? Please rate it again on a scale from 0 to 100. |
| 37 | Closing remarks | If your mood has changed, that’s a sign that you've successfully organized your thoughts. That's it for today. Good job! Feel free to reach out anytime. |
