# Supplementary figures and images for: Adapting the Number of Questions Based on Detected Psychological Distress for Cognitive Behavioral Therapy With an Embodied Conversational Agent: Comparative Study
Source: JMIR Form Res. 2024 Mar 14;8:e50056. doi: 10.2196/50056 (PMC10979340; doi:10.2196/50056)

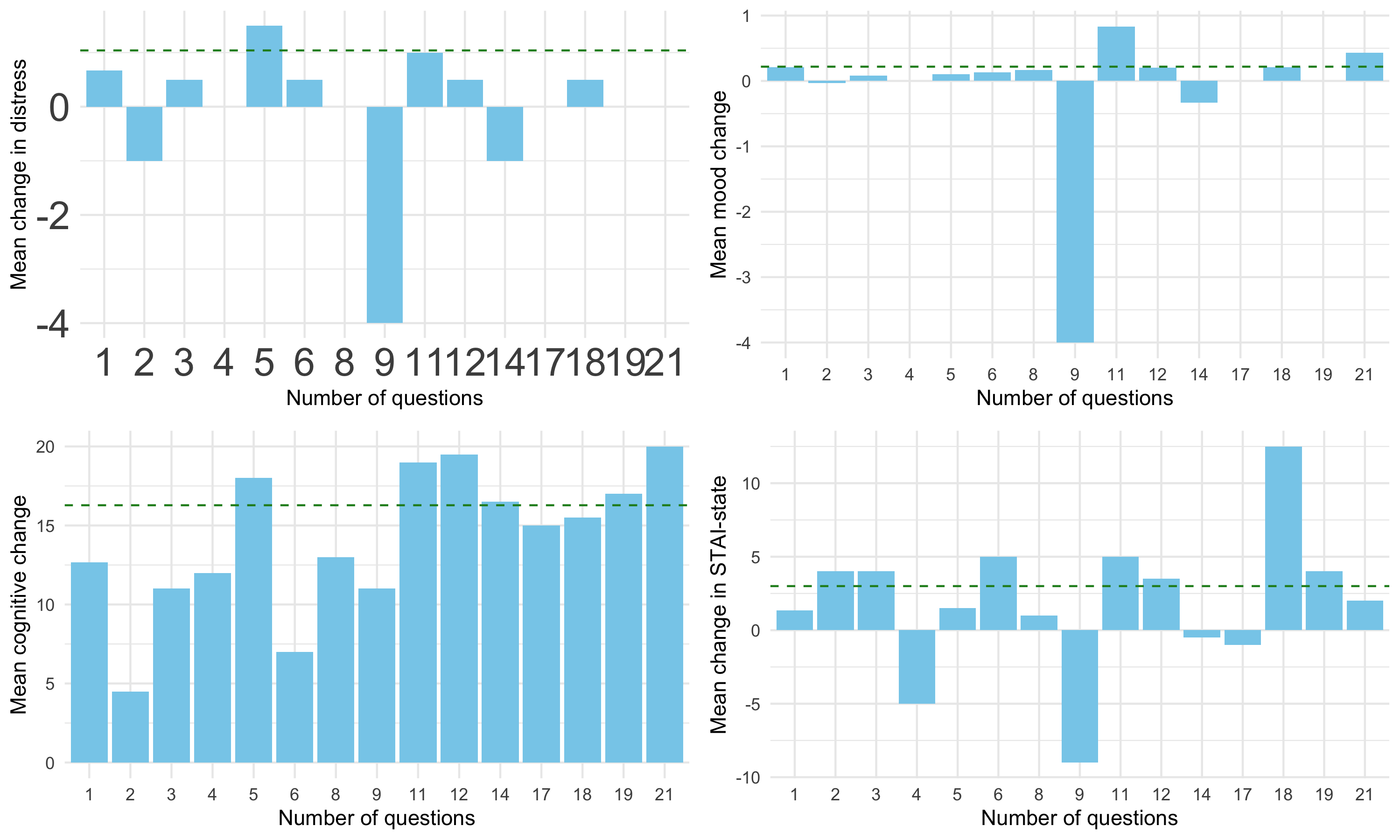

Supplement: Multimedia Appendix 2 [file formative_v8i1e50056_app2.png]

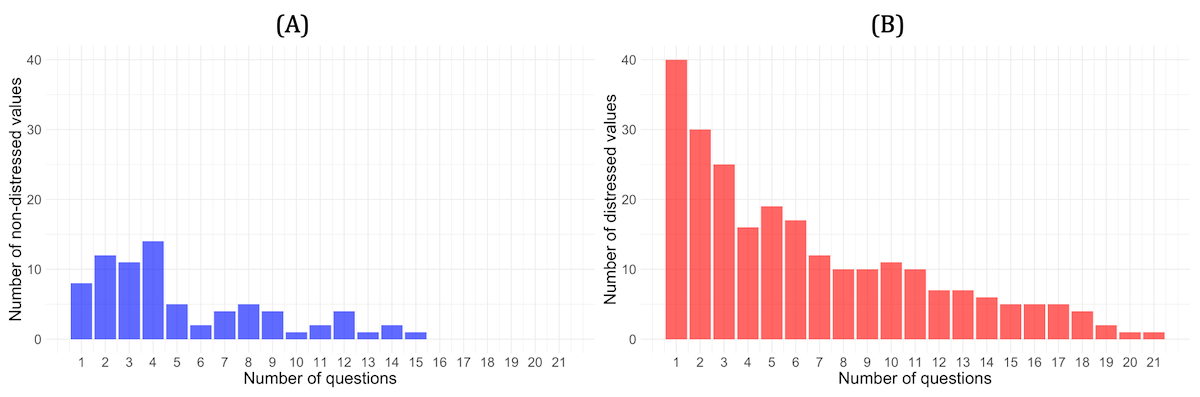

Supplement: Multimedia Appendix 3 [file formative_v8i1e50056_app3.png]
